# Genetic interactions of schizophrenia using gene-based statistical epistasis exclusively identify nervous system-related pathways and key hub genes

**DOI:** 10.3389/fgene.2023.1301150

**Published:** 2024-01-08

**Authors:** Sathish Periyasamy, Pierre Youssef, Sujit John, Rangaswamy Thara, Bryan J. Mowry

**Affiliations:** ^1^ Queensland Brain Institute, The University of Queensland, Brisbane, QLD, Australia; ^2^ Queensland Centre for Mental Health Research, The Park Centre for Mental Health, Wacol, QLD, Australia; ^3^ Schizophrenia Research Foundation, Chennai, Tamil Nadu, India

**Keywords:** epistasis, schizophrenia, genetic interaction analysis, GWAS, hub genes, systems biology

## Abstract

**Background:** The relationship between genotype and phenotype is governed by numerous genetic interactions (GIs), and the mapping of GI networks is of interest for two main reasons: 1) By modelling biological robustness, GIs provide a powerful opportunity to infer compensatory biological mechanisms via the identification of functional relationships between genes, which is of interest for biological discovery and translational research. Biological systems have evolved to compensate for genetic (i.e., variations and mutations) and environmental (i.e., drug efficacy) perturbations by exploiting compensatory relationships between genes, pathways and biological processes; 2) GI facilitates the identification of the direction (alleviating or aggravating interactions) and magnitude of epistatic interactions that influence the phenotypic outcome. The generation of GIs for human diseases is impossible using experimental biology approaches such as systematic deletion analysis. Moreover, the generation of disease-specific GIs has never been undertaken in humans.

**Methods:** We used our Indian schizophrenia case-control (case-816, controls-900) genetic dataset to implement the workflow. Standard GWAS sample quality control procedure was followed. We used the imputed genetic data to increase the SNP coverage to analyse epistatic interactions across the genome comprehensively. Using the odds ratio (OR), we identified the GIs that increase or decrease the risk of a disease phenotype. The SNP-based epistatic results were transformed into gene-based epistatic results.

**Results:** We have developed a novel approach by conducting gene-based statistical epistatic analysis using an Indian schizophrenia case-control genetic dataset and transforming these results to infer GIs that increase the risk of schizophrenia. There were ∼9.5 million GIs with a *p*-value ≤ 1 × 10^−5^. Approximately 4.8 million GIs showed an increased risk (OR > 1.0), while ∼4.75 million GIs had a decreased risk (OR <1.0) for schizophrenia.

**Conclusion:** Unlike model organisms, this approach is specifically viable in humans due to the availability of abundant disease-specific genome-wide genotype datasets. The study exclusively identified brain/nervous system-related processes, affirming the findings. This computational approach fills a critical gap by generating practically non-existent heritable disease-specific human GIs from human genetic data. These novel datasets can train innovative deep-learning models, potentially surpassing the limitations of conventional GWAS.

## 1 Introduction

Schizophrenia, a debilitating mental disorder with a lifetime prevalence of around 1% and notable morbidity and mortality, presents a significant public health challenge. Currently, the Psychiatric Genomics Consortium (PGC) has identified over 250 schizophrenia risk loci, among more than 300 unique association loci linked to major psychiatric disorders, necessitating urgent exploration (Lam et al., 2019; Periyasamy et al., 2019; Ripke et al., 2020). The discovery of these loci has been greatly facilitated by Genome-wide Association Studies (GWAS), a cornerstone in the field, having successfully unveiled numerous loci associated with various complex traits and diseases (Visscher et al., 2017). The GWAS Catalogue of September 2023 (Marees et al., 2018) catalogues a remarkable 552,116 significant variant-trait associations from 6,566 publications. Despite GWAS’s remarkable success in identifying this multitude of loci, only a relatively small fraction of single nucleotide polymorphisms (SNPs) associated with diseases/traits have been functionally characterised. This limitation stems from diverse challenges encompassing scientific, technical, methodological, and funding aspects. Additionally, a significant majority of low penetrance risk variants identified by GWAS are situated in non-coding regions, impacting gene regulation, thereby presenting substantial methodological hurdles in accurately pinpointing the causal variant, identifying the genes it influences, and deducing the molecular mechanisms of diseases (Egervari et al., 2018). The unravelling of disease mechanisms and gaining fundamental biological insights are pivotal for offering improved evidence-based and personalised treatments. Existing *in vivo* models need increased sensitivity to observe phenotypes associated with low penetrance risk variants, given that the biological robustness inherent in these systems can complicate results. Consequently, novel approaches are imperative for swiftly translating GWAS discoveries into viable medical interventions.

The complexity of biological systems raises many scientific challenges as they have evolved to be robust in the face of environmental and genetic perturbations. Systemic properties such as extreme-polygenicity (Boyle et al., 2017), pleiotropy and robustness are a result of redundant and compensatory mechanisms that have evolved between molecular and organismic resolutions (Periyasamy et al., 2009; Periyasamy et al., 2013), contributing to nonadditive/nonlinear effects in biological systems. These compensatory mechanisms regulated by various molecular activities can contribute to biological epistasis (Moore, 2005; Sackton and Hartl, 2016). Epistasis/GI manifests when the effects of mutations/variants depend on the genetic background on which they occur (Domingo et al., 2019). Such dependency on genetic background/context can cause phenotypic diversity between individuals and, on evolutionary timescales, incompatibilities between different species. Such issues raise concern when using *in-vivo* models to represent humans, as evidenced by the alarming drug failure rates (∼96%) when translating from preclinical to clinical trials (Hingorani et al., 2019). Technologies that generate data to study the above systemic properties are required to bridge the genotype-phenotype gap. Fortunately, some high-throughput technologies in genomics could be advantageous to infer GIs, where they are thought to be one of the causes of missing heritability (Eichler et al., 2010; Zuk et al., 2012). GIs frequently connect genes between compensatory pathways, so they confound our ability to predict phenotype from gene-environment interactions. This major challenge must be addressed to realise the potential of precision medicine. Causalities can be modelled by identifying GIs that enhance or suppress a phenotype (Costanzo et al., 2016). Currently, there is a yeast GI (Costanzo et al., 2016) and a database containing ∼223,000 GIs for the human cancer cell lines (Horlbeck et al., 2018; Mair et al., 2019), a mere fraction of the estimated number of gene-gene combinations (∼200 million) for humans. These datasets were generated by observing cellular phenotypes for many combinations of double gene-silencing experiments, analogous to generating gene-based statistical epistasis data. However, this laborious experimental approach becomes an improbable task when considering human endophenotypes, let alone human disease phenotypes such as schizophrenia. Hence, the only solution will be to use *in silico* approaches. Although there are computational approaches to predicting GIs (Sun et al., 2014; Wei et al., 2014; Niel et al., 2015; Fang et al., 2019), genome-wide disease-specific human-level GI data have not been generated to date.

## 2 Methods

### 2.1 Sample collection

All participants provided written informed consent, and the study received ethical approval from each participating institution’s institutional review boards. This research adhered to the Strengthening the Reporting of Genetic Association Studies (STREGA) reporting guideline. Our participant cohort was drawn from The Schizophrenia Research Foundation in Chennai, India, and overwhelmingly comprised individuals of Tamil ethnicity, accounting for more than 97% of the sample. We employed standardised assessment tools, which were administered in Tamil when necessary. These tools included the Diagnostic Interview for Genetic Studies (Nurnberger et al., 1994), the Family Interview for Genetic Studies (Maxwell, 1992), the DSM-IV diagnostic criteria, and a consensus diagnostic procedure.

The study population encompassed two main groups: 1) a family dataset that was assembled based on the presence of multiple affected family members and 2) a case-control dataset. Our study involved 1,716 participants, of which 816 were diagnosed with schizophrenia. The recruitment, genotyping, and data analysis were carried out in consecutive phases, commencing on 1 January 2001.

To estimate the study power, we used a dedicated R package powerGWASInteraction (Kooperberg and LeBlanc, 2008) to conduct power analysis for genetic interactions. We used the default parameters for the GxG interaction model and conducted the analysis using powerGG (). The power was set at 80%, alpha at 0.05, case-control ratio at 0.5, disease prevalence at 0.01 and the number of genes tested was set to 20,000. The sample size was estimated to be approximately 8,000. In contrast, the false discovery rate (FDR) method is widely utilised in situations with numerous tests where the expense of false positives is minimal and employing the Bonferroni procedure is unfeasible due to the sheer volume of simultaneous tests (i.e., ∼200 million in this case). It maintains strict control over the family-wise error rate and becomes more conservative as the number of comparisons increases. This means that as the number of tests grows, Bonferroni correction becomes increasingly stringent, potentially leading to a higher likelihood of false negatives. FDR, on the other hand, focuses on the proportion of false positives among the declared significant findings. It controls the rate of false discoveries and, in many cases, allows for a higher discovery rate than Bonferroni, especially in situations with many hypotheses being tested. Hence, we also used the r package pwrFDR (Jung, 2005; Liu and Hwang, 2007), a dedicated application to calculate power based on FDR. The average power was 100% for an effect size of 1.5, a sample size of 1716 and an alpha of 0.05.

### 2.2 Genotyping and quality control

The study sample was assembled and underwent genotyping in sequential phases spanning 15 years. The family sample, consisting of 658 individuals, was subjected to genotyping utilising the Illumina CNV370 Beadchip array. Concurrently, a group of unrelated controls, totalling 199 individuals, was initially enrolled in the study and subsequently genotyped using Illumina OmniExpress-12 arrays. In the later stages of the study, additional cases and controls were brought on board. They underwent genotyping in two distinct batches: the first wave included 1,370 individuals, and the second encompassed 1,008 individuals. It is important to note that the figures provided here represent the initial sample sizes before the quality control process. The standard GWAS sample quality control procedures have been followed to check for ancestry and relatedness (Peterson et al., 2019).

#### 2.2.1 MDS analysis

**FIGURE 1:**
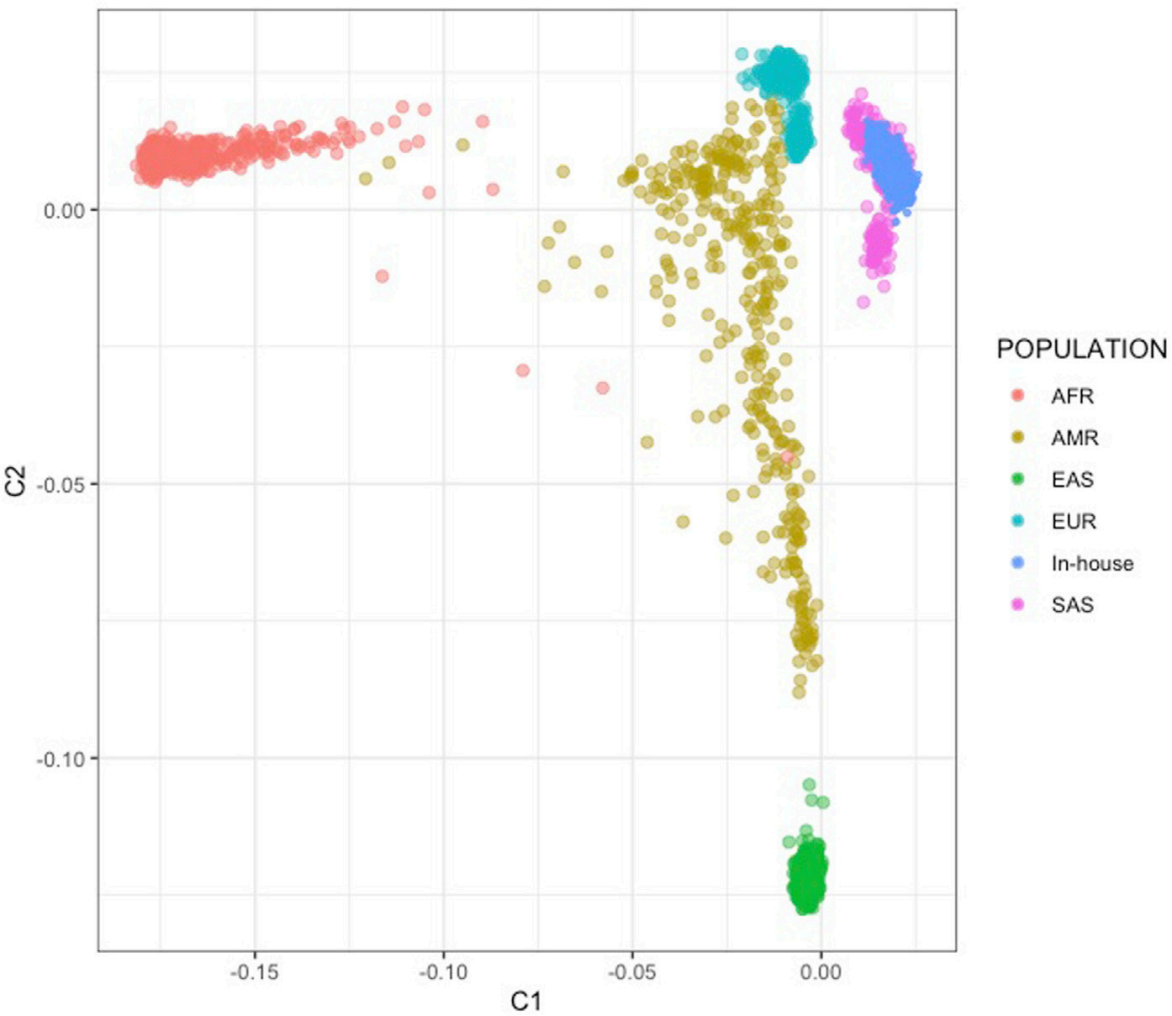
MDS analysis using 1,000G SAS population. Super populations include African (AFR), Admixed American (AMR), European (EUR), East Asian (EAS) and South Asian (SAS). The figure shows the Indian case-control datasets (In-House) clustering with the SAS population.

We conducted a multi-dimensional scaling (MDS) analysis with the objectives of assessing potential array-related effects and determining the genetic similarity of the Indian samples to the South Asian (SAS) population in the 1,000 Genomes (1 KG) phase 3 reference dataset. The MDS analysis was performed using all common SNPs found in both the Indian family and case-control datasets and the 1,000 Genomes dataset, comprising a total of 2,504 samples and 26,231 SNPs after pruning. We excluded SNPs with ambiguous strand information to avoid any potential issues related to strand orientation. The absence of outliers in the analysis confirmed that our samples exhibited Indian ancestry (See Figure 1).

### 2.3 Genotype imputation

#### 2.3.1 Pre-imputation QC

To ensure the identification of genetically related individuals, we compiled a set of 26,939 single nucleotide polymorphisms (SNPs) that were in linkage disequilibrium independently and had a minor allele frequency exceeding 0.05. These SNPs were consistently present across all genotyping platforms employed in the study. In the family samples, we employed identity by descent (IBD) analysis and cross-referenced the results with the clinical records associated with the four datasets. This allowed us to detect and eliminate duplicate entries and incorrectly specified relationships. Individuals demonstrating relatedness beyond second-degree (as indicated by PI-HAT≥0.1875) were excluded from the case-control datasets. Subsequently, we integrated the four datasets to identify discrepancies in relatedness within and between families. This process led to the removal of 143 individuals who exhibited such discrepancies. Before proceeding with the imputation process, the cleaned datasets underwent further filtering based on specific parameters, including --geno 0.02, --maf 0.01, --hwe 0.001, and --mind 0.1.

#### 2.3.2 Imputation

As previously described, we applied imputation to each of our four datasets using the 1,000 Genomes (1 KG) phase 3 reference dataset. During this process, we updated the SNP coordinates to align with human genome build 37 and ensured they were oriented on the positive strand. To achieve accurate phasing, we employed SHAPEIT v2.72720. Subsequently, we carried out an imputation using IMPUTE v2.3.021. For the imputation procedure, each chromosome was divided into segments of 5 megabases with a 250-kilobase overlap. As recommended by the authors, we utilised all available 1 KG populations, encompassing approximately 2,504 individuals, as the reference dataset. The resulting imputed data was then converted into ‘best guess’ genotypes and saved in binary PLINK format for further analysis.

#### 2.3.3 Post-imputation QC

From an initial set of 81,177,102 imputed single nucleotide polymorphisms (SNPs), we applied a series of stringent filters and quality control measures to reduce the dataset to approximately 6.2–6.5 million SNPs. These filters included: 1) Removing SNPs with an INFO score less than 0.8. 2) Extracting of unrelated individuals. 3) Eliminating SNPs with a missing data rate exceeding 0.05. 4) Discarding SNPs with a Hardy-Weinberg equilibrium (HWE) *p*-value less than 1 × 10^−5^ in both cases and controls. 5) Applying a stricter HWE threshold, with a *p*-value less than 1 × 10^−6^ in controls. 6) Employing an even more stringent HWE threshold, with a *p*-value less than 1 × 10^−10^ in cases. After applying these quality control criteria, a total of 5.5 million genetic variants and 1716 individuals (comprising 816 cases and 900 controls) remained in the dataset, having successfully met all filtering and quality control requirements.

### 2.4 Generating GI data

**FIGURE 2:**
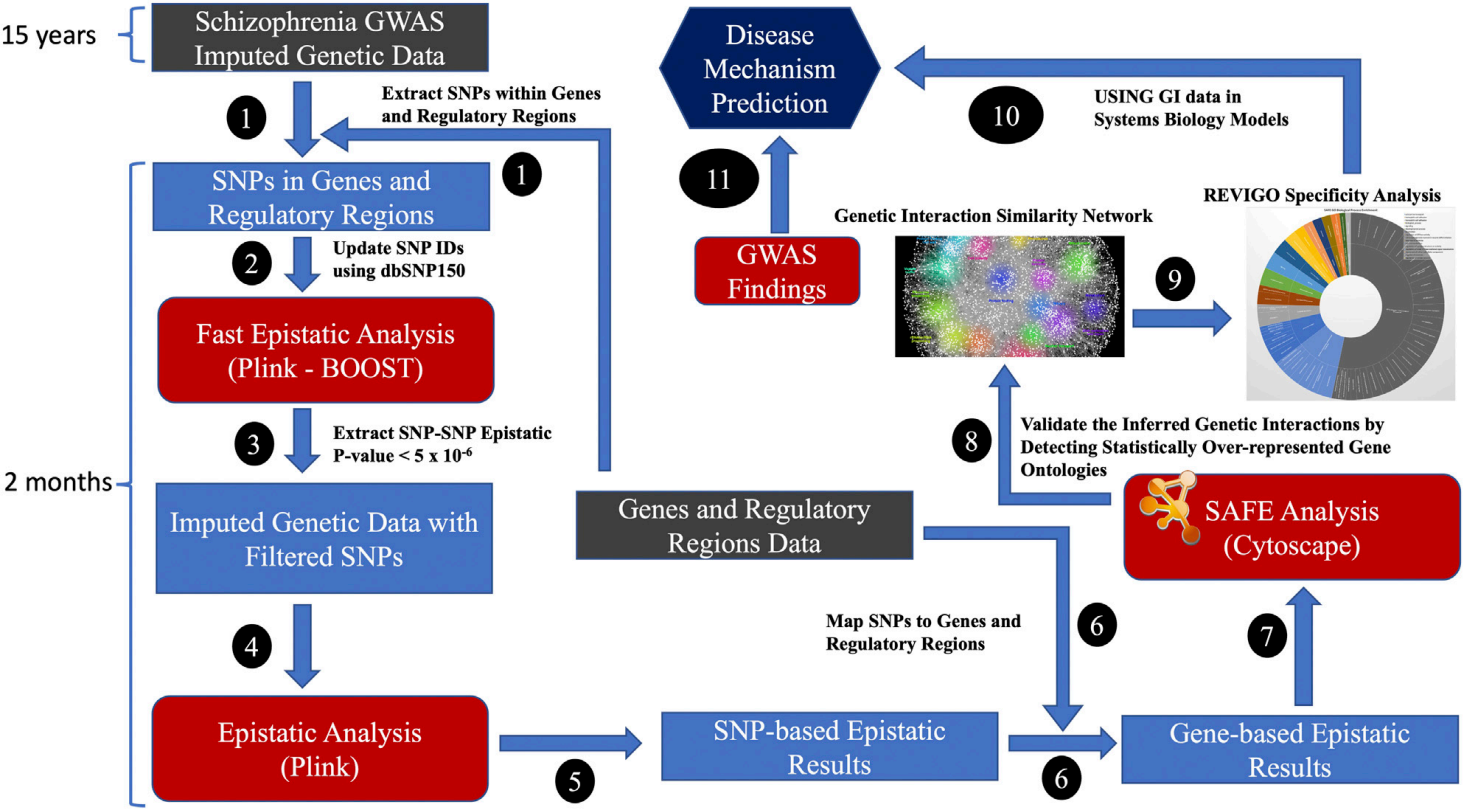
The high-performance computing (HPC) bioinformatics workflow for generating GIs from disease-specific case-control datasets; Genes and regulatory regions data: FANTOM5 and GENCODE project.

Imputed data increased the SNP coverage for comprehensive analysis of epistatic interactions across the genome. We mapped the genes with promoter regions from the FANTOM5 project (https://fantom.gsc.riken.jp/) (Lizio et al., 2015; Noguchi et al., 2017), which had robust experimentally validated promotor regions for GENCODE genes. All SNPs within the promotor and their genes, considered to represent a gene, were included in the analysis. Only intergenic SNP-SNP interactions were considered for generating GIs. The generation of GIs using the standard epistatic analysis method required significant investment in time (i.e., many weeks), computational and storage resources as it generated results for billions of SNP-SNP combinations. Initially, we used the fast epistatic analysis method, BOOST (Wan et al., 2010), on the SNPs located in genes and their regulatory regions to identify the most relevant SNPs and then used the standard epistatic analysis method on nominally significant epistatic SNP-SNP combinations to generate OR for GIs (Steps 1-6 in Figure 2). Plink 1.9 (Purcell et al., 2007) was used to conduct genome-wide epistasis analysis. The multiple testing burden was further reduced by collapsing the *p*-values and ORs of SNP-SNP to gene-gene combinations. The SNP-based epistatic results were transformed into gene-based epistatic results. The most significant SNP-SNP *p*-value combination representing the two genes was transformed into *p*-values representing the gene-gene combinations. By using the odds ratio (OR), we identified the GIs that increase (OR >1) or decrease (OR <1) the risk of a disease phenotype (i.e., schizophrenia). The number of gene-gene combinations for multiple tests was estimated to be ∼200 million, assuming 20,000 protein-coding genes. The Bonferroni threshold was set at 2.5 × 10^−10^ (i.e. 0.05/200,000,000), and the false discovery rate threshold (FDR value of 0.05) for significant GIs was set at 2.375 × 10^−3^ for FDR significant GIs, assuming 200 million gene-gene combinations.

### 2.5 Validating GI data

A subset of GIs with OR>2 and OR<0.5 was validated using spatial analysis of functional enrichment (SAFE) (Baryshnikova, 2016; Baryshnikova et al., 2018) and REVIGO’s (Supek et al., 2011) GO disease specificity analyses to confirm the relevance of the generated data (Steps 7–9). SAFE is a systematic method for annotating biological networks and examining functional organisation. Due to the hierarchical nature of GO biological processes, the resulting lists of GO terms can be large and highly redundant. REVIGO summarises the non-redundant GO terms using a semantic similarity algorithm that uses the ‘most informative common ancestor’ approach to identify precise GO biological process terms. We applied SAFE to annotate the schizophrenia GI similarity network with GO terms and used REVIGO to validate the GIs of schizophrenia. We used REVIGO to reduce redundancy and summarise incomprehensible lists of Gene Ontology terms by finding a representative subset of the terms by clustering using semantic similarity measures. We used GOATOOLS (https://github.com/tanghaibao/goatools) (Klopfenstein et al., 2018) to plot the GO biological process hierarchy. GO release 2023-07-27 (http://current.geneontology.org/ontology/go-basic.obo) was used for this analysis.

### 2.6 Identifying hub genes

We considered all intragenic genetic interactions with nominal *p*-values (1 × 10^−5^) to identify the hub genes and the potential drives of schizophrenia. We considered a two-fold increase or decrease in ORs for this analysis. We considered OR > 2 for all increased-risk GIs, and for all decreased-risk GIs, we considered OR<0.5. From this list, we tabulated the genetic interactions that showed an increased risk for schizophrenia and decreased or no risk for schizophrenia.

### 2.7 Developing genetic interaction resource for schizophrenia

We have generated a global schizophrenia GI dataset comprising ∼9.5 million GIs (1 × 10^−5^). These data include intragenic and as well as promoter/enhancer regions.

## 3 Results

We have developed a novel approach by conducting gene-based statistical epistatic analysis using an Indian schizophrenia case-control (case-816, controls-900) genetic dataset and transforming these results to infer GIs. Although underpowered, we successfully generated meaningful GI data with a small sample size 1716. The SAFE and REVIGO analysis confirmed this by identifying brain/nervous system-related biological processes exclusively. None of the GIs reached the Bonferroni threshold of 2.5 × 10^−10^. However, all 9.5 million GIs surpassed the FDR significance threshold.

### 3.1 Genetic interactions that increase the risk of schizophrenia

There were ∼9.5 million GIs with a nominal *p*-value ≤ 1 × 10^−5^. Approximately 4.8 million GIs showed an increased risk (Odds Ratio >1.0), while ∼4.75 million GIs had decreased or no risk (Odds Ratio <1.0) for schizophrenia. This dataset includes interactions between representative SNPs located in genes and SNPs overlapping multiple genes, including promoters. There were 15 GIs with OR >8. When considering GIs involved between intragenic regions, there were ∼125,000 GIs with a nominal *p*-value ≤ 1 × 10^−5^. This will be the first step in developing a global GI resource for schizophrenia.

### 3.2 Biological processes involved in schizophrenia

**FIGURE 3:**
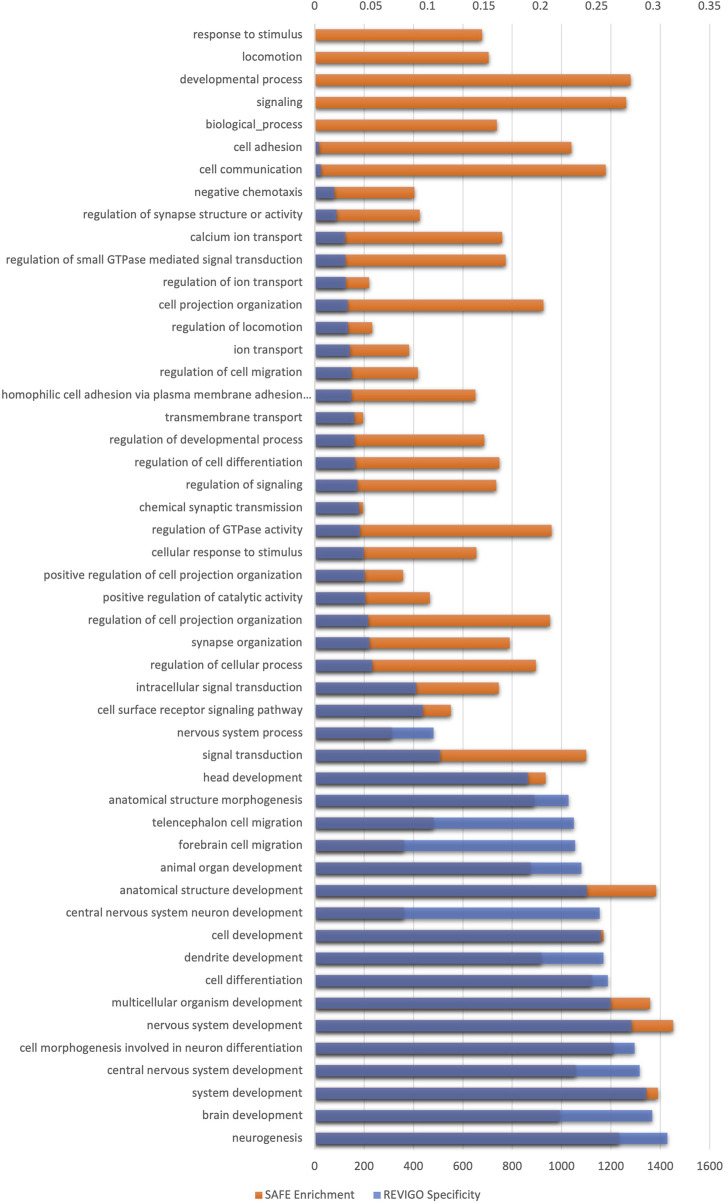
Spatial analysis of functional enrichment of GI data generated using the Indian schizophrenia genetic data. The *x*-axis on the bottom shows the enrichment score of biological processes, and the *x*-axis on the top shows the specificity of biological processes. The *y*-axis shows the GO biological process enriched after multiple testing corrections. The biological processes are organised in the ascending order of specificity.

There were ∼110,000 GIs with OR >2 or OR <0.5. SAFE and REVIGO analysis exclusively identified brain/nervous system-related biological processes, affirming the findings. The genetic interactions of schizophrenia were functionally enriched in brain/nervous system-related biological processes. Most biological processes were semantically related to nervous system development, with synaptic transmission being the next largest cluster, followed by synapse organisation. Figure 3 shows the enriched biological processes identified by SAFE. The lowest processes in the GO hierarchy are all related to the nervous system (see Supplementary Figure S1).

### 3.3 Hub genes of schizophrenia

**FIGURE 4:**
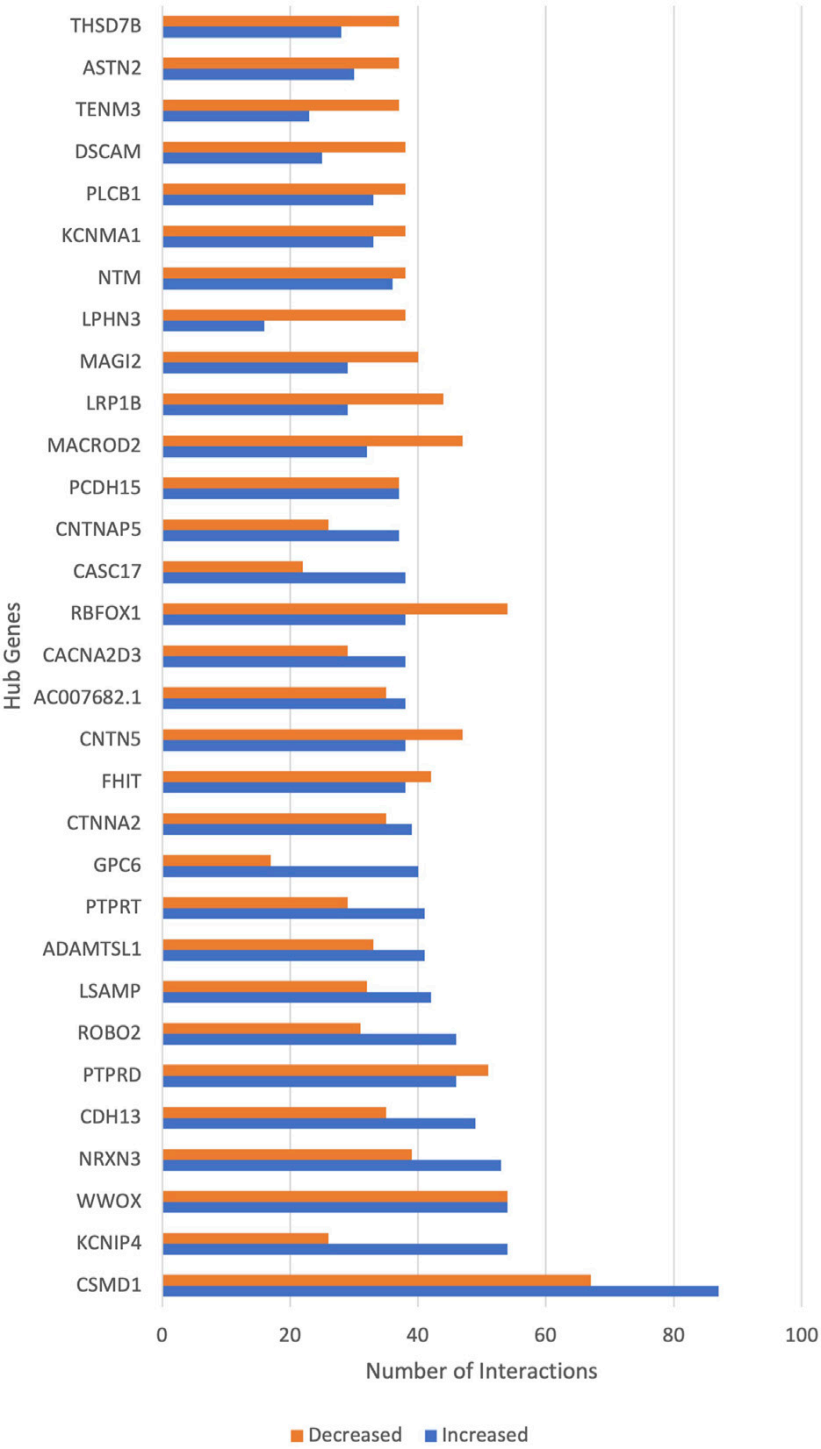
The hub genes of schizophrenia. The *x*-axis shows the number of gene interactions of hub genes that increase or decrease the risk of schizophrenia. The *y*-axis shows the hub genes of schizophrenia.

Notable genes are hub genes, which can be considered drivers of schizophrenia. Figure 4 shows the top hub genes involved in schizophrenia. *CSMD1* had the highest GIs, followed by *KCNIP4*, *WWOX* and *NRXN3*. Each hub gene increases the risk for schizophrenia when interacting with a subset of genes and shows decreased or no risk for schizophrenia with another subset of genes. Supplementary Table S1 shows the complete list of hub genes and their functions based on existing literature.

## 4 Discussion

We observed the nervous system development to have the highest enrichment, and neurogenesis to have the highest specificity. The schizophrenia GIs were primarily enriched in biological processes involved in the nervous system and neuron-related processes. In contrast to model organisms, this approach is specifically viable in humans due to the availability of abundant disease-specific genome-wide genotype datasets. Despite limited power, meaningful GI data was generated with a small sample. SAFE and REVIGO analyses exclusively identified brain/nervous system-related processes, affirming the reliability of the generated GIs. This computational approach fills a critical gap by generating practically non-existent heritable disease-specific human GIs from human genetic data. These novel datasets can train innovative deep-learning models for post-GWAS functional characterisation, potentially surpassing the limitations of conventional GWAS.

### 4.1 A novel computational approach to generate practically non-existent GIs from human genetic data

Biologists have used double gene silencing approaches for over two decades to study GIs (Mani et al., 2008). Generating GI data has been daunting as it involves establishing cell/animal lines to observe relevant phenotypes, which often takes weeks to months per GI experiment. However, this laborious experimental approach becomes an improbable task when considering human endophenotypes, let alone complex human disease phenotypes such as schizophrenia. Moreover, this is neither ethical nor practical in humans, and observational studies investigating phenotypes in any organism are subject to a range of unknown confounding effects that are difficult to control. We have proposed a novel approach by conducting analogous gene-based statistical epistatic analysis using human genetic data and transforming these results to infer gene-based GIs. This approach is only feasible in humans because, unlike model organisms, abundant GWAS data are available, including large-scale publicly available biobank data. Compared to traditional observational experiments, our strategy is robust to confounding, rapid, and cost-effective and can be performed using existing GWAS data. The novel contribution of our approach consists in how we generate and utilise the GI information. OR is an often-ignored parameter. This information is useful in detecting the direction and magnitude of GIs. While existing *in silico* approaches focus on the presence or absence of GIs, we also include the effect size and direction of disease-specific GIs to infer increased or decreased disease risk.

### 4.2 There currently needs to be comprehensive heritable disease-specific human GI data

Only two cellular-level GI datasets are available for the research community: a wet-lab generated comprehensive yeast GI dataset (∼8 million GIs) and a cancer GI dataset (∼230,000) generated using human cancer cell lines with cell growth/viability as the phenotype. Our approach addresses this problem by developing a novel *in silico* method to generate heritable disease-specific GIs such as schizophrenia. The significant short-term outcomes will include: 1) Producing a schizophrenia GI resource for the global biomedical science community. 2) A contribution to the modelling of biological robustness, thereby providing a powerful opportunity to infer compensatory biological mechanisms and to study genetic (i.e., variations and mutations) and environmental (i.e., drug efficacy) perturbations. 3) The generation of GIs for other human heritable diseases. In addition to contributing to basic research in schizophrenia, disease-specific GIs are highly important for translational research. The ubiquitous nature of biological robustness is a major problem for pharmaceutical drug discovery, as humans can develop resistance to therapeutic agents (Boucher and Jenna, 2013). GIs can map the elusive relationship between genotype and phenotype and are thought to underlie many common heritable diseases. Furthermore, exploring GI networks may help to realise the gene networks underlying phenotypical variation and polygenic diseases.

### 4.3 A GI dataset for post-GWAS functional characterisation

While GWAS has successfully unveiled numerous risk loci, it faces ongoing critique due to its limited ability to elucidate many molecular mechanisms influencing human phenotypes that contribute to disease risk. GWAS loci often prove challenging to decipher because linkage disequilibrium frequently masks the causal variant during association, and identifying the specific gene responsible for mediating variant effects on the trait is seldom achievable solely through GWAS data. Moreover, a significant proportion of GWAS risk variants with low penetrance reside in non-coding regions that impact the gene regulation (Egervari et al., 2018), presenting considerable methodological hurdles in accurately pinpointing the causal variant, determining the genes it governs, and deducing disease molecular mechanisms (Schaid et al., 2018). These obstacles are unique to variant-trait associations within the realm of GWAS. However, our approach remains unhindered by these challenges as it can directly predict the causal genes and molecular mechanisms from human genetic data, aligning with one of GWAS’s primary objectives—to identify causal genes, molecular mechanisms, and potential interventions.

### 4.4 Future directions

We plan to use the workflow to generate GIs for a larger dataset, such as the PGC dataset. We anticipate large datasets could reveal more significant GIs for schizophrenia. These datasets can be used for various systems biology studies, such as developing explainable deep learning models to predict disease mechanisms (Ma et al., 2018).

## Data Availability

The original contributions presented in the study are publicly available. This data can be found here: https://espace.library.uq.edu.au/view/UQ:24c3e5e.

## Supplementary material

SUPPLEMENTARY FIGURE S1 The Gene Ontology biological processes enriched in schizophrenia. The yellow nodes indicate the enriched process, and the white nodes indicate the processes linking the enriched nodes to higher- level biological processes.

SUPPLEMENTARY TABLE S1 Tabs one and two show the number of hub gene interactions that increase or decrease the risk of schizophrenia. Tab three contains the list of top hup genes and their functions.

## References

[B1] Baryshnikova A. (2016). Systematic functional annotation and visualization of biological networks. Cell Syst. 2 (6), 412–421. 10.1016/j.cels.2016.04.014 27237738

[B2] Baryshnikova A. (2018). Spatial analysis of functional enrichment (SAFE) in large biological networks. Methods Mol. Biol. 1819, 249–268. 10.1007/978-1-4939-8618-7_12 30421408

[B3] Boucher B. Jenna S. (2013). Genetic interaction networks: better understand to better predict. Front. Genet. 4 (290), 290. 10.3389/fgene.2013.00290 24381582 PMC3865423

[B4] Boyle E. A. Li Y. I. Pritchard J. K. (2017). An expanded view of complex traits: from polygenic to omnigenic. Cell 169 (7), 1177–1186. 10.1016/j.cell.2017.05.038 28622505 PMC5536862

[B5] Costanzo M. VanderSluis B. Koch E. N. Baryshnikova A. Pons C. Tan G. (2016). A global genetic interaction network maps a wiring diagram of cellular function. Science 353 (6306), aaf1420. 10.1126/science.aaf1420 27708008 PMC5661885

[B6] Domingo J. Baeza-Centurion P. Lehner B. (2019). The causes and consequences of genetic interactions (epistasis). Annu. Rev. Genomics Hum. Genet. 20 (1), 433–460. 10.1146/annurev-genom-083118-014857 31082279

[B7] Egervari G. Kozlenkov A. Dracheva S. Hurd Y. L. (2018). Molecular windows into the human brain for psychiatric disorders. Mol. Psychiatry 24, 653–673. 10.1038/s41380-018-0125-2 29955163 PMC6310674

[B8] Eichler E. E. Flint J. Gibson G. Kong A. Leal S. M. Moore J. H. (2010). Missing heritability and strategies for finding the underlying causes of complex disease. Nat. Rev. Genet. 11 (6), 446–450. 10.1038/nrg2809 20479774 PMC2942068

[B9] Fang G. Wang W. Paunic V. Heydari H. Costanzo M. Liu X. (2019). Discovering genetic interactions bridging pathways in genome-wide association studies. Nat. Commun. 10 (1), 4274. 10.1038/s41467-019-12131-7 31537791 PMC6753138

[B10] Hingorani A. D. Kuan V. Finan C. Kruger F. A. Gaulton A. Chopade S. (2019). Improving the odds of drug development success through human genomics: modelling study. Sci. Rep. 9 (1), 18911. 10.1038/s41598-019-54849-w 31827124 PMC6906499

[B11] Horlbeck M. A. Xu A. Wang M. Bennett N. K. Park C. Y. Bogdanoff D. (2018). Mapping the genetic landscape of human cells. Cell 174 (4), 953–967. 10.1016/j.cell.2018.06.010 30033366 PMC6426455

[B12] Jung S.-H. (2005). Sample size for FDR-control in microarray data analysis. Bioinformatics 21 (14), 3097–3104. 10.1093/bioinformatics/bti456 15845654

[B13] Klopfenstein D. V. Zhang L. Pedersen B. S. Ramírez F. Warwick Vesztrocy A. Naldi A. (2018). GOATOOLS: a Python library for Gene Ontology analyses. Sci. Rep. 8 (1), 10872. 10.1038/s41598-018-28948-z 30022098 PMC6052049

[B14] Kooperberg C. LeBlanc M. (2008). Increasing the power of identifying gene x gene interactions in genome-wide association studies. Genet. Epidemiol. 32 (3), 255–263. 10.1002/gepi.20300 18200600 PMC2955421

[B15] Lam M. Chen C.-Y. Li Z. Martin A. R. Bryois J. Ma X. (2019). Comparative genetic architectures of schizophrenia in East Asian and European populations. Nat. Genet. 51 (12), 1670–1678. 10.1038/s41588-019-0512-x 31740837 PMC6885121

[B16] Liu P. Hwang J. T. G. (2007). Quick calculation for sample size while controlling false discovery rate with application to microarray analysis. Bioinformatics 23 (6), 739–746. 10.1093/bioinformatics/btl664 17237060

[B17] Lizio M. Harshbarger J. Shimoji H. Severin J. Kasukawa T. Sahin S. (2015). Gateways to the FANTOM5 promoter level mammalian expression atlas. Genome Biol. 16 (1), 22. 10.1186/s13059-014-0560-6 25723102 PMC4310165

[B18] Ma J. Yu M. K. Fong S. Ono K. Sage E. Demchak B. (2018). Using deep learning to model the hierarchical structure and function of a cell. Nat. Methods 15, 290–298. 10.1038/nmeth.4627 29505029 PMC5882547

[B19] Mair B. Moffat J. Boone C. Andrews B. J. (2019). Genetic interaction networks in cancer cells. Curr. Opin. Genet. Dev. 54, 64–72. 10.1016/j.gde.2019.03.002 30974317 PMC6820710

[B20] Mani R. Onge R. P.St. Hartman J. L. Giaever G. Roth F. P. (2008). Defining genetic interaction. Proc. Natl. Acad. Sci. 105 (9), 3461–3466. 10.1073/pnas.0712255105 18305163 PMC2265146

[B21] Marees A. T. de Kluiver H. Stringer S. Vorspan F. Curis E. Marie-Claire C. (2018). A tutorial on conducting genome-wide association studies: quality control and statistical analysis. Int. J. Methods Psychiatric Res. 27 (2), e1608. 10.1002/mpr.1608 PMC6001694 29484742

[B22] Maxwell M. (1992). Family Interview for genetic studies (FIGS): manual for FIGS. Bethesda, MD: National Institute of Mental Health.

[B23] Moore J. H. (2005). A global view of epistasis. Nat. Genet. 37, 13–14. 10.1038/ng0105-13 15624016

[B24] Niel C. Sinoquet C. Dina C. Rocheleau G. (2015). A survey about methods dedicated to epistasis detection. Front. Genet. 6, 285. 10.3389/fgene.2015.00285 26442103 PMC4564769

[B25] Noguchi S. Arakawa T. Fukuda S. Furuno M. Hasegawa A. Hori F. (2017). FANTOM5 CAGE profiles of human and mouse samples. Sci. Data 4 (1), 170112. 10.1038/sdata.2017.112 28850106 PMC5574368

[B26] Nurnberger J. I. Jr Blehar M. C. Kaufmann C. A. York-Cooler C. Simpson S. G. Harkavy-Friedman J. (1994). Diagnostic Interview for genetic studies: rationale, unique features, and training. Archives General Psychiatry 51 (11), 849–859. 10.1001/archpsyc.1994.03950110009002 7944874

[B27] Periyasamy S. Gray A. Kille P. (2009). “Multiscale adaptive dynamics from molecules to cells,” in Foundations of systems biology in engineering (Denver, Colorado, USA: Omnipress).

[B28] Periyasamy S. Gray A. Kille P. (2013). The bottom-up approach to defining life: deciphering the functional organization of biological cells via multi-objective representation of biological complexity from molecules to cells. Front. Physiology 4, 369. 10.3389/fphys.2013.00369 PMC3866382 24385968

[B29] Periyasamy S. John S. Padmavati R. Rajendren P. Thirunavukkarasu P. Gratten J. (2019). Association of schizophrenia risk with disordered niacin metabolism in an Indian genome-wide association study. JAMA Psychiatry 76 (10), 1026–1034. 10.1001/jamapsychiatry.2019.1335 31268507 PMC6613304

[B30] Peterson R. E. Kuchenbaecker K. Walters R. K. Chen C.-Y. Popejoy A. B. Periyasamy S. (2019). Genome-wide association studies in ancestrally diverse populations: opportunities, methods, pitfalls, and recommendations. Cell 179 (3), 589–603. 10.1016/j.cell.2019.08.051 31607513 PMC6939869

[B31] Purcell S. Neale B. Todd-Brown K. Thomas L. Ferreira M. A. Bender D. (2007). PLINK: a tool set for whole-genome association and population-based linkage analyses. Am. J. Hum. Genet. 81 (3), 559–575. 10.1086/519795 17701901 PMC1950838

[B32] Ripke S. Walters J. T. O’Donovan M. C. (2020). Mapping genomic loci prioritises genes and implicates synaptic biology in schizophrenia. medRxiv.

[B33] Sackton T. B. Hartl D. L. (2016). Genotypic context and epistasis in individuals and populations. Cell 166 (2), 279–287. 10.1016/j.cell.2016.06.047 27419868 PMC4948997

[B34] Schaid D. J. Chen W. Larson N. B. (2018). From genome-wide associations to candidate causal variants by statistical fine-mapping. Nat. Rev. Genet. 19 (8), 491–504. 10.1038/s41576-018-0016-z 29844615 PMC6050137

[B35] Sun X. Lu Q. Mukherjee S. Crane P. K. Elston R. Ritchie M. D. (2014). Analysis pipeline for the epistasis search - statistical versus biological filtering. Front. Genet. 5, 106. 10.3389/fgene.2014.00106 24817878 PMC4012196

[B36] Supek F. Bošnjak M. Škunca N. Šmuc T. (2011). REVIGO summarizes and visualizes long lists of gene Ontology terms. PLOS ONE 6 (7), e21800. 10.1371/journal.pone.0021800 21789182 PMC3138752

[B37] Visscher P. M. Wray N. R. Zhang Q. Sklar P. McCarthy M. I. Brown M. A. (2017). 10 years of GWAS discovery: biology, function, and translation. Am. J. Hum. Genet. 101 (1), 5–22. 10.1016/j.ajhg.2017.06.005 28686856 PMC5501872

[B38] Wan X. Yang C. Yang Q. Xue H. Fan X. Tang N. L. S. (2010). BOOST: a fast approach to detecting gene-gene interactions in genome-wide case-control studies. Am. J. Hum. Genet. 87 (3), 325–340. 10.1016/j.ajhg.2010.07.021 20817139 PMC2933337

[B39] Wei W.-H. Hemani G. Haley C. S. (2014). Detecting epistasis in human complex traits. Nat. Rev. Genet. 15, 722–733. 10.1038/nrg3747 25200660

[B40] Zuk O. Hechter E. Sunyaev S. R. Lander E. S. (2012). The mystery of missing heritability: genetic interactions create phantom heritability. Proc. Natl. Acad. Sci. 109 (4), 1193–1198. 10.1073/pnas.1119675109 22223662 PMC3268279

