# Supplementary material for: Genetic interactions of schizophrenia using gene-based statistical epistasis exclusively identify nervous system-related pathways and key hub genes
Source: Front Genet. 2024 Jan 8;14:1301150. doi: 10.3389/fgene.2023.1301150 (PMC10800577; doi:10.3389/fgene.2023.1301150)
Supplement: Supplementary file 2 [file Image1.pdf]

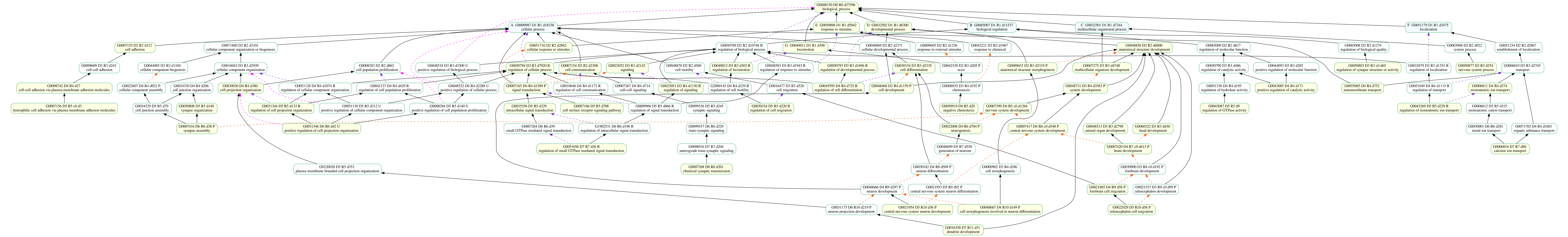

G0008150 D0 R0 d27596
biological_process
A G0009987 D1 R1 d18338
cellular process
E G0050896 D1 R1 d5042
response to stimulus
D G0032502 D1 R1 d6300
developmental process
B G0065007 D1 R1 d11537
biological regulation
C G0032501 D1 R1 d7344
multicellular organismal process
F G0051179 D1 R1 d3475
localization
G0007155 D2 R2 d312
cell adhesion
G0071840 D2 R2 d3101
cellular component organization or biogenesis
G0051716 D2 R2 d2862
cellular response to stimulus
G0050789 D2 R2 d10748 R
regulation of biological process
G G0040011 D1 R1 d398
locomotion
G0048869 D2 R2 d2371
cellular developmental process
G0009605 D2 R2 d1236
response to external stimulus
G0042221 D2 R2 d1967
response to chemical
G0048856 D2 R2 d6000
anatomical structure development
G0065009 D2 R2 d817
regulation of molecular function
G0065008 D2 R2 d1274
regulation of biological quality
G0003008 D2 R2 d922
system process
G0051234 D2 R2 d2867
establishment of localization
G0098609 D3 R3 d243
cell-cell adhesion
G0044085 D3 R3 d1104
cellular component biogenesis
G0016043 D3 R3 d2939
cellular component organization
G0008283 D2 R2 d862
cell population proliferation
G0048518 D3 R3 d3300 U
positive regulation of biological process
G0050794 D3 R3 d7920 R
regulation of cellular process
G0007154 D2 R2 d2308
cell communication
G0023052 D3 R3 d2142
signaling
G0048870 D2 R2 d569
cell motility
G0040012 D3 R3 d303 R
regulation of locomotion
G0048583 D3 R3 d1943 R
regulation of response to stimulus
G0050793 D3 R3 d1696 R
regulation of developmental process
G0030154 D3 R3 d2335
cell differentiation
G0042330 D2 R3 d205 P
taxis
G0009653 D2 R3 d2319 P
anatomical structure morphogenesis
G0007275 D3 R3 d4748
multicellular organism development
G0050790 D3 R3 d486
regulation of catalytic activity
G0044093 D3 R3 d285
positive regulation of molecular function
G0050803 D3 R3 c0 d65
regulation of synapse structure or activity
G0032879 D3 R3 d1353 R
regulation of localization
G0050877 D3 R3 d254
nervous system process
G0006810 D3 R3 d2745
transport
G0098742 D4 R4 d27
cell-cell adhesion via plasma-membrane adhesion molecules
G0022607 D4 R4 d922 P
cellular component assembly
G0034330 D4 R4 d206
cell junction organization
G0030030 D4 R4 d380
cell projection organization
G0051128 D4 R4 d1074 R
regulation of cellular component organization
G0042127 D4 R4 d429 R
regulation of cell population proliferation
G0048522 D4 R4 d2288 U
positive regulation of cellular process
G0007165 D4 R4 d1589 P
signal transduction
G0010646 D4 R4 d1172 R
regulation of cell communication
G0007267 D4 R4 d714
cell-cell signaling
G0023051 D3 R4 d1130 R
regulation of signaling
G2000145 D4 R4 d239 R
regulation of cell motility
G0016477 D3 R3 d520
cell migration
G0045595 D4 R4 d725 R
regulation of cell differentiation
G0048468 D3 R4 d1159 P
cell development
G0006935 D3 R4 d192 P
chemotaxis
G0048731 D3 R4 d3583 P
system development
G0051336 D4 R4 d195
regulation of hydrolase activity
G0043085 D4 R4 d171
positive regulation of catalytic activity
G0055085 D4 R4 d752
transmembrane transport
G0051049 D4 R4 d1115 R
regulation of transport
G0006811 D4 R4 d374
monoatomic ion transport
G0007156 D5 R5 c0 d3
homophilic cell adhesion via plasma membrane adhesion molecules
G0034329 D5 R5 d70
cell junction assembly
G0050808 D5 R5 d140
synapse organization
G0031344 D5 R5 d133 R
regulation of cell projection organization
G0051130 D5 R5 d312 U
positive regulation of cellular component organization
G0008284 D5 R5 d140 U
positive regulation of cell population proliferation
G0035556 D5 R5 d329
intracellular signal transduction
G0007166 D5 R5 d708
cell surface receptor signaling pathway
G0009966 D5 R5 d866 R
regulation of signal transduction
G0099536 D5 R5 d245
synaptic signaling
G0030334 D5 R5 d220 R
regulation of cell migration
G0050919 D4 R5 d20
negative chemotaxis
G0007399 D4 R5 c0 d1264
nervous system development
G0043087 D5 R5 d9
regulation of GTPase activity
G0043269 D5 R5 d229 R
regulation of monoatomic ion transport
G0006812 D5 R5 d315
monoatomic cation transport
G0007416 D6 R6 d38 P
synapse assembly
G0031346 D6 R6 d42 U
positive regulation of cell projection organization
G0007264 D6 R6 d39
small GTPase mediated signal transduction
G1902531 D6 R6 d198 R
regulation of intracellular signal transduction
G0099537 D6 R6 d229
trans-synaptic signaling
G0022008 D4 R6 d704 P
neurogenesis
G0007417 D4 R6 c0 d548 P
central nervous system development
G0048513 D3 R3 d2798
animal organ development
G0060322 D3 R3 d430
head development
G0030001 D6 R6 d281
metal ion transport
G0071702 D4 R4 d1601
organic substance transport
G0051056 D7 R7 d26 R
regulation of small GTPase mediated signal transduction
G0098916 D7 R7 d204
anterograde trans-synaptic signaling
G0048699 D5 R7 d558
generation of neurons
G0007420 D4 R7 c0 d413 P
brain development
G0006816 D7 R7 d96
calcium ion transport
G0120036 D5 R5 d353
plasma membrane bounded cell projection organization
G0007268 D8 R8 d201
chemical synaptic transmission
G0030182 D4 R8 d509 P
neuron differentiation
G0000902 D3 R4 d286
cell morphogenesis
G0030900 D3 R8 c0 d192 P
forebrain development
G0048666 D4 R9 d297 P
neuron development
G0021953 D5 R9 d92 P
central nervous system neuron differentiation
G0021885 D4 R9 d58 P
forebrain cell migration
G0021537 D3 R9 c0 d99 P
telencephalon development
G0031175 D6 R10 d219 P
neuron projection development
G0021954 D5 R10 d36 P
central nervous system neuron development
G0048667 D4 R10 d149 P
cell morphogenesis involved in neuron differentiation
G0022029 D5 R10 d56 P
telencephalon cell migration
G0016358 D7 R11 d31
dendrite development
